# Effect of assist-as-needed robotic gait training on the gait pattern post stroke: a randomized controlled trial

**DOI:** 10.1186/s12984-020-00800-4

**Published:** 2021-02-05

**Authors:** J. F. Alingh, B. M. Fleerkotte, B. E. Groen, J. S. Rietman, V. Weerdesteyn, E. H. F. van Asseldonk, A. C. H. Geurts, J. H. Buurke

**Affiliations:** 1 Sint Maartenskliniek Research, PO Box 9011, 6500 GM Nijmegen, The Netherlands; 2 Donders Institute for Brain, Cognition and Behaviour, Department of Rehabilitation, Radboud University Medical Center, Nijmegen, The Netherlands; 3 Roessingh Research and Development, Enschede, The Netherlands; 4 Roessingh Center for Rehabilitation, Enschede, The Netherlands; 5 Department of Biomechanical Engineering, University of Twente, Enschede, The Netherlands; 6 Department of Biomedical Signals and Systems, University of Twente, Enschede, The Netherlands

**Keywords:** Stroke, Rehabilitation, Gait, Robotics, Quality, External work

## Abstract

**Background:**

Regaining gait capacity is an important rehabilitation goal post stroke. Compared to clinically available robotic gait trainers, robots with an assist-as-needed approach and multiple degrees of freedom (AAN_mDOF_) are expected to support motor learning, and might improve the post-stroke gait pattern. However, their benefits compared to conventional gait training have not yet been shown in a randomized controlled trial (RCT). The aim of this two-center, assessor-blinded, RCT was to compare the effect of AAN_mDOF_ robotic to conventional training on the gait pattern and functional gait tasks during post-stroke inpatient rehabilitation.

**Methods:**

Thirty-four participants with unilateral, supratentorial stroke were enrolled (< 10 weeks post onset, Functional Ambulation Categories 3–5) and randomly assigned to six weeks of AAN_mDOF_ robotic (combination of training in LOPES-II and conventional gait training) or conventional gait training (30 min, 3–5 times a week), focused on pre-defined training goals. Randomization and allocation to training group were carried out by an independent researcher. External mechanical work (W_EXT_), spatiotemporal gait parameters, gait kinematics related to pre-defined training goals, and functional gait tasks were assessed before training (T0), after training (T1), and at 4-months follow-up (T2).

**Results:**

Two participants, one in each group, were excluded from analysis because of discontinued participation after T0, leaving 32 participants (AAN_mDOF_ robotic n = 17; conventional n = 15) for intention-to-treat analysis. In both groups, W_EXT_ had decreased at T1 and had become similar to baseline at T2, while gait speed had increased at both assessments. In both groups, most spatiotemporal gait parameters and functional gait tasks had improved at T1 and T2. Except for step width (T0–T1) and paretic step length (T0–T2), there were no significant group differences at T1 or T2 compared to T0. In participants with a pre-defined goal aimed at foot clearance, paretic knee flexion improved more in the AAN_mDOF_ robotic group compared to the conventional group (T0–T2).

**Conclusions:**

Generally, AAN_mDOF_ robotic training was not superior to conventional training for improving gait pattern in subacute stroke survivors. Both groups improved their mechanical gait efficiency. Yet, AAN_mDOF_ robotic training might be more effective to improve specific post-stroke gait abnormalities such as reduced knee flexion during swing.

*Trial registration* Registry number Netherlands Trial Register (www.trialregister.nl): NTR5060. Registered 13 February 2015.

## Introduction

Regaining gait capacity is one of the most reported rehabilitation goals post stroke [1–3]. Besides basic gait independence and the ability to adapt gait to environmental demands, rehabilitation is often focused on optimizing the individual gait pattern, particularly in the early phase post stroke. After unilateral supratentorial stroke, the hemiparetic gait pattern is commonly characterized by pes equinovarus during swing and/or loading [4], knee instability during early and/or midstance [5, 6], impaired ankle plantarflexion power during push-off [4], and reduced knee flexion during (pre)swing of the paretic leg [5]. As a consequence, asymmetry in step length [5] and/or single support time are observed in many patients with post-stroke hemiparesis [7]. In addition, hemiparetic gait is associated with reduced gait speed [8], increased fall risk [9], and limited community ambulation [10]. Hence, improving the post-stroke gait pattern is an important rehabilitation goal.

Robotic gait training has the potential to improve the post-stroke gait pattern [11–17], but its benefits compared to conventional gait training are still under debate [11–18]. Most clinically available robotic gait trainers lack the ability to adjust the robotic actuation based on the user’s performance, which may restrain motor learning [18]. In contrast, robotic gait trainers with a so called ‘assist-as-needed’ (AAN) approach adapt guidance to the user’s needs [19, 20] and allow support of specific subtasks of the gait cycle [20], thereby promoting active involvement of the user and, thus, motor learning [21–23]. Furthermore, robotic gait trainers with ample degrees of freedom allow a (near) normal gait pattern, in particular with respect to active balance control during walking [21, 24]. In addition, sufficient allowance of movement variability optimizes the amount of error information needed for motor learning [25]. Consequently, robotic gait training with AAN principles and multiple degrees of freedom (AAN_mDOF_) has the potential to improve gait post stroke. However, no evidence from randomized controlled trials is yet available for its superiority compared to conventional gait training, in particular with regard to the gait pattern, during primary inpatient stroke rehabilitation.

As nearly all kinematic gait deviations and/or spatiotemporal gait abnormalities are translated into irregular movements of the body center of mass of the body (COM), we evaluated the quality of the post-stroke gait pattern based on the COM trajectory. COM movement relative to its surroundings is represented by external mechanical work (W_EXT_) [26]. In healthy individuals who walk at their preferred speed, COM movements in directions other than the walking direction are typically minimized [27], and W_EXT_ is relatively small. Stroke survivors, however, often show compensatory movements in the frontal, sagittal and/or transversal planes while walking, resulting in irregular and enlarged COM trajectories [28] and increased W_EXT_ [29], reflecting a reduced quality of the gait pattern. As increased gait speed is generally associated with increased W_EXT_ [30, 31], interpretation of changes in W_EXT_ should be related to changes in gait speed.

The primary aim of the present study was to evaluate whether six weeks AAN_mDOF_ robotic gait training would be superior to conventional gait training in terms of W_EXT_ in stroke survivors during their inpatient rehabilitation. A secondary aim was to evaluate whether this effect would be retained four months after the intervention. We hypothesized that, given a similar increase in gait speed between groups, the increase in W_EXT_ would be smaller following robotic training compared to conventional training one week and four months after the intervention period. A third aim was to evaluate the AAN_mDOF_ robotic gait training on spatiotemporal gait parameters, kinematics related to pre-defined training goals, and functional gait tasks.

## Methods

### Participants

Stroke survivors admitted for inpatient rehabilitation to two rehabilitation centers in the Netherlands (Sint Maartenskliniek, Nijmegen; Roessingh Center for Rehabilitation, Enschede) were assessed for eligibility by their treating rehabilitation physician or physical therapist from October 2015 until June 2019. Inclusion criteria were: (1) adult (≥ 18 years of age) after a first or recurrent unilateral ischemic or hemorrhagic supratentorial stroke (< 10 weeks post onset), (2) impairment of one or more prerequisites of gait according to Gage et al. [32]. Exclusion criteria were: (1) inability to walk without support, with or without supervision (Functional Ambulation Category (FAC) 0–2), (2) medical conditions interfering with gait, (3) inability to understand verbal instructions, (4) severe visual problems e.g. hemianopia or visuospatial neglect, (5) no independent ambulation prior to stroke, (6) depressed mood assessed with the Hospital Anxiety and Depression Scale (HADS > 7), (7) severe lower limb spasticity (at any level) assessed with the Modified Ashworth Scale (MAS ≥ 3), (8) severe lower limb contracture (at any level) determined by a physical examination, (9) body weight ≥ 140 kg, (10) skin problems at any body site where the support harness or straps of the robotic gait trainer were to be fitted, and (11) expected length of stay in rehabilitation center < 6 weeks. Exclusion criteria 7 to 10 were applied primarily to prevent inappropriate or unsafe fitting of the robotic gait trainer. Individuals who were eligible and willing to participate received study information from the researcher. All participants gave written informed consent before definitive inclusion, in accordance with the Declaration of Helsinki. Demographic and clinical characteristics were collected: sex (male/female), height (cm), hemiparetic side (left/right), use of ankle–foot orthosis (yes/no), lower limb motor impairment (Fugl Meyer Assessment [33]—leg score; 0–34), lower limb strength (Motricity Index [34]—leg score; 0–100), cognition (Montreal Cognitive Assessment (MoCA [35]; 0–30), and communication skills (Utrechts Communicatie Onderzoek (UCO [36]—subscale conversation; 1–5).

### Study design and randomization

**Fig. 1:**
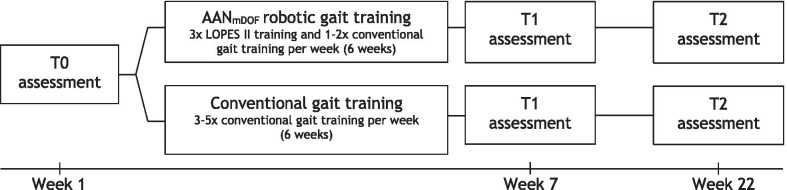
Study design

This study was conducted as a two-center, assessor-blinded, randomized controlled, parallel group trial. The study protocol (NL 50748.044.14) was approved by the Medical Ethical Committee Twente (Enschede, the Netherlands) and registered in the Netherlands Trial Register (NTR5060). Figure 1 provides an overview of the study design. Assessments were performed before (T0), within one week after (T1), and four months after (T2) the six-week intervention period. At each center, all assessments were performed by one assessor who was blinded for group allocation. After completing the T0 assessment, a stratified block randomization with an allocation ratio of 1:1 was used. Participants were stratified by baseline gait speed (≤ 0.4 m/s or  > 0.4 m/s) and allocated to the AAN_mDOF_ robotic or conventional gait training groups using random permuted blocks (block sizes two and four) within each strata. An independent researcher generated the random allocation sequence, transferred it to numbered envelopes, and handed the envelope to the participant to inform about the group allocation after completing the T0 assessment.

### Intervention

Prior to the start of the training an individual training goal was selected by a rehabilitation physician based on clinical examination. The pre-defined training goals were derived from the kinematic aspects of the prerequisites of gait defined by Gage et al. [32] and were operationalized as improving: foot clearance (swing), knee stability (stance), limb loading (stance), or foot prepositioning (swing). The AAN_mDOF_ robotic gait training group received three 30-min sessions of individually tailored LOPES II training per week. LOPES II is a treadmill-based AAN_mDOF_ robotic gait trainer, combined with a body-weight support system (MOOG BV, Nieuw-Vennep, the Netherlands). LOPES II has eight powered degrees of freedom, actuating pelvic translations in the anterior/posterior and lateral directions, hip flexion/extension, hip adduction/abduction, and knee flexion/extension. Ankle dorsiflexion movements can be supported using toe-lifters or conventional ankle–foot orthoses. For a detailed description of the LOPES II see Meuleman et al. [20]. At the start of the training, individually-tailored, minimal levels of body-weight support and general and specific guidance forces were determined at which the participant was just able to match the reference gait trajectories, related to the pre-defined training goal, of the LOPES II. Across the training sessions, the goal was to match the reference gait trajectories of the LOPES II, while gradually reducing the level of body-weight support, reducing the general and specific guidance forces, and increasing the gait speed. Real-time feedback about the participant’s gait pattern was provided by the user interface of LOPES II, complemented by verbal feedback from the treating physical therapist. AAN_mDOF_ robotic gait training was complemented with a maximum of two 30-min individual gait training sessions per week, according to the latest insights in neurorehabilitation [37]. Thus, when using the term AAN_mDOF_ robotic gait training in the remainder of this text, this refers to a combination of robotic gait training in LOPES II and conventional therapy. The training frequency and LOPES II settings were documented in a logbook.

The conventional training group received three to five 30-min individual gait training sessions per week, according to the latest insights in neurorehabilitation [37]. Physical therapists provided verbal feedback about the participant’s performance with emphasis on attainment of the individual primary training goal. The training frequency was documented in a logbook. Both the AAN_mDOF_ robotic and conventional gait training group could receive group training as part of their regular gait rehabilitation program, in addition to the scheduled individual gait training sessions per week. The training frequency of the group sessions was documented in a logbook. Use of interactive treadmill or other robotic gait trainers was not allowed during the intervention period. After the end of the intervention period (after the T1 assessment), participants were allowed to continue their regular (inpatient or outpatient) rehabilitation program, but these gait training sessions were no longer logged.

### Procedure

During each assessment a 3D-gait analysis was performed. Reflective markers (n = 39) were attached to the participant according to the Plug-In-Gait Full Body model (Plug-In-Gait, Vicon Motion Systems Ltd, Oxford, UK). Marker positions were recorded by infrared cameras (f_s_ = 100 Hz; Vicon mX 1.7.1, Oxford Metrics, UK). Participants were instructed to walk at their self-selected speed along a straight 6-m walkway. Participants wore their own shoes and were allowed to use an ankle–foot orthosis if necessary, which could vary between assessments as a consequence of motor recovery. Use of other walking aids was not allowed. At least 15 strides were collected during each assessment. Data was analyzed using custom written software (MATLAB, Mathworks Inc, Natick, MA, USA). Initial contact and foot-off were determined with the velocity-based algorithm as described by Zeni et al. [38].

### Outcomes

#### Primary outcome measure

The primary outcome W_EXT_ was determined per stride through analysis of the energy changes at the level of the COM relative to the surroundings [26]. The energy level of the body (E_EXT_) is determined by the sum of potential and kinetic energy of the COM per stride:

$$E_{EXT} = \frac{1}{2}MV_{forward}^{2} + \left( {MgH + \frac{1}{2}MV_{vertical}^{2} } \right) + \frac{1}{2}MV_{lateral}^{2}$$

where *M* is the total body mass (kg), *g* is gravity (m/s^2^), and *H* and *V* are the height (m) and velocity in the forward, vertical and lateral direction (m/s) of the COM relative to the surrounding. W_EXT_ is defined as the sum of the increments of the E_EXT_ curve per stride. W_EXT_ was normalized for body mass and stride length (J/kg/m). As W_EXT_ is associated with walking velocity [30, 31], W_EXT_ is always reported together with gait speed.

### Secondary outcome measures

The following spatiotemporal parameters were calculated using the marker data collected from each trial of the 3D-gait analysis: gait speed (m/s), step width (m), step length (m), and single-support time (% gait cycle). Symmetry ratios were calculated for step length and single-support time, and expressed as the absolute difference from 0.5 (perfect symmetry), according to the following equation:

$$Symmetry ratio = \left| { 0.5 - \frac{{Parameter_{paretic leg} }}{{Parameter_{non - paretic leg} + Parameter_{paretic leg} }} } \right|$$

In addition, the following functional gait tasks and clinical leg motor scores were recorded during each assessment: 6-Minute Walk Test [39], 10-Meter Walk Test [40], Timed Up and Go Test [41], Functional Gait Assessment [42], Fugl Meyer Assessment [33]—leg score, and Motricity Index [34]—leg score. Participants were allowed to use an ankle–foot orthosis and/or a walking aid during the functional gait tasks when necessary.

### Individual training goals

To evaluate the training effects on the pre-defined training goals, Vicon Plug-In-Gait model and software were used to calculate the individual gait kinematics per stride. Foot clearance, knee stability in stance (reduction in knee extension thrust), limb loading and foot prepositioning were evaluated by maximal knee flexion of the paretic leg during early swing, the difference in maximal knee extension velocity between the paretic and non-paretic leg during single-support phase, single-support time symmetry, and minimal knee flexion of the paretic leg during terminal swing, respectively.

### Power calculation

Power analysis performed using STATA version 10.1 showed that a sample size of 50 participants (α = 0.05, β = 0.10, including 10% drop-out) was sufficient to demonstrate a group difference in W_EXT_ of 0.13 J/kg/m after the intervention [43].

### Statistical analysis

All statistical analyses were performed using SPSS statistics version 19 (IBM SPSS Statistics, Chicago, USA). Baseline characteristics were compared between groups using independent t-tests or Mann–Whitney U tests for continuous variables, and chi-square tests for categorical variables. W_EXT_, spatiotemporal parameters, and gait kinematics were averaged per individual over all strides per assessment (T0–T2). Effects of the intervention at T1 and T2 on primary and secondary outcomes were separately analyzed, according to an ‘intention-to-treat’ principle, using linear mixed model for repeated measures with a fixed effect for Group (AAN_mDOF_ robotic vs conventional) and Time (T0 vs T1, or T0 vs T2). All linear mixed models used a restricted maximum likelihood estimation to obtain the results, an unstructured covariance matrix, and Šidák adjustment for multiple testing. Effects of the intervention on the pre-defined training goals were analyzed per subgroup of participants with the same pre-defined training goal (n ≥ 10), according to an ‘per-protocol’ analysis, using non-parametric Mann–Whitney U tests on difference scores for each outcome (T0 vs T1, or T0 vs T2). The significance level was set at p < 0.05 for all tests.

## Results

**Fig. 2:**
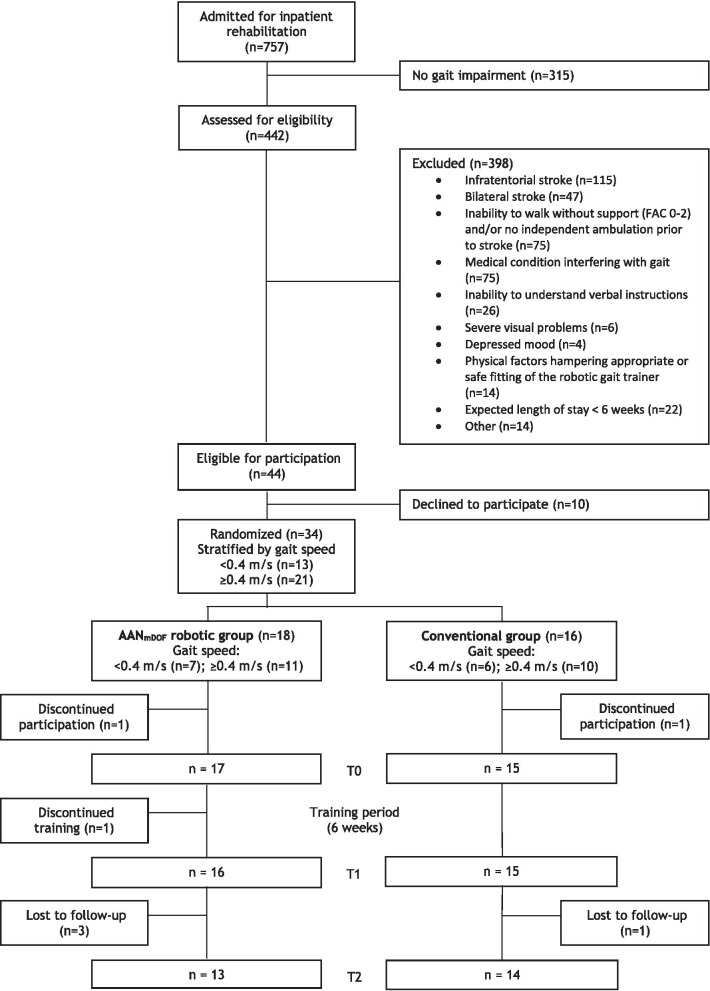
CONSORT Flowchart

The participants’ flow is presented in Fig. 2. Recruitment started in October 2015 and was stopped in June 2019 due to end of funding. Thirty-four individuals were randomly assigned to the AAN_mDOF_ robotic (n = 18; gait speed < 0.4 m/s, n = 7) or conventional gait training group (n = 16; gait speed < 0.4 m/s, n = 6). Two participants, one in each group, discontinued participation directly after T0, because they expected the study protocol to be too physically demanding. Hence 32 participant were included in the intention-to-treat analysis (AAN_mDOF_ robotic n = 17; conventional n = 15). One participant discontinued the robotic gait training, because the study protocol was too physically demanding. Another four subjects (AAN_mDOF_ robotic n = 3; conventional n = 1) were lost to follow-up after the post-intervention assessment, because of time requirements (n = 2) or medical reasons unrelated to the study (n = 2). Baseline demographic and clinical characteristics, and individual training goals did not differ between groups (see Table 1).

**Table 1:** Baseline demographic, clinical characteristics, and individual training goals for the AAN_mDOF_ robotic and conventional gait training groups (mean ± SD or number)

	AAN_mDOF_ robotic n = 17	Conventional n = 15
Sex, male/female (n)	10/7	10/5
Age (years)	60.6 ± 9.3	56.8 ± 9.8
Height (cm)	177.4 ± 7.6	177.7 ± 7.5
Weight (kg)	80.8 ± 16.0	79.3 ± 14.3
Type of stroke, ischemic/haemorrhagic (n)	13/4	11/4
Time since stroke (wks)	5.4 ± 1.8	5.9 ± 2.1
Hemiparetic side, left/right (n)	7/10	7/8
Use of ankle–foot orthosis (n)	8	6
FAC score (n)		
3	10	6
4	6	8
5	1	1
Fugl Meyer Assessment—leg score	24.2 ± 4.6	23.4 ± 6.8
Motricity Index—leg score	63.9 ± 17.0	62.5 ± 26.4
HADS—subscale depression	1.8 ± 1.5	1.2 ± 0.9
MoCA	24.1 ± 4.2	23.4 ± 4.1
UCO—subscale conversation (n)		
4	0	2
5	17	13
Individual training goal (n)		
Foot clearance	6	6
Knee stability	6	7
Limb loading	4	2
Foot prepositioning	1	0

*FAC score* Functional Ambulation Category (range 0–5), Fugl Meyer Assessment-leg score (range 0–34), Motricity Index leg score (range 0–100), *HADS* Hospital Anxiety and Depression Scale-subscale depression (range 0–21), *MoCA* Montreal Cognitive Assessment (range 0–30), *UCO* Utrechts Communicatie Onderzoek-subscale conversation (range 1–5)

### Details of interventions and adverse effects

In the robotic training group, one participant discontinued training after 2 sessions, whereas the other participants received a median of 15 (interquartile range (IQR): 13.8–15.3) individual robotic gait training sessions. In accordance with the training protocol, a reduction in average body weight support (week 1: 8.3 ± 5.6%; week 6: 6.7 ± 3.5%), general guidance force (week 1: 61.1 ± 22.0%; week 6: 22.2 ± 25.6%), and specific guidance force (week 1: 42.8 ± 21.8%; week 6: 25.0 ± 23.2%) was applied, while average gait speed was increased (week 1: 1.60 ± 0.51 km/h; week 6: 2.40 ± 0.62 km/h) across the robotic training sessions. In addition to the robotic gait training, participants in this group received a median of 11 (IQR: 7.5–12.0) individual and 6 (IQR: 4.8–10.5) group sessions of conventional gait training, resulting in a total median number of 32 (IQR: 26.0–37.8) training sessions during the intervention period. The conventional training group received a median of 18 (IQR: 14.5–22.0) individual and 9 (IQR: 7.5–12.0) group sessions of conventional gait training, resulting in a total median number of 27 (IQR: 22.0–34.0) training sessions during the intervention period. One participant experienced a fall with wheelchair, outside the study context, but was able to continue conventional gait training after one week of rest. No additional adverse events were reported.

### External mechanical work and gait speed

**Fig. 3:**
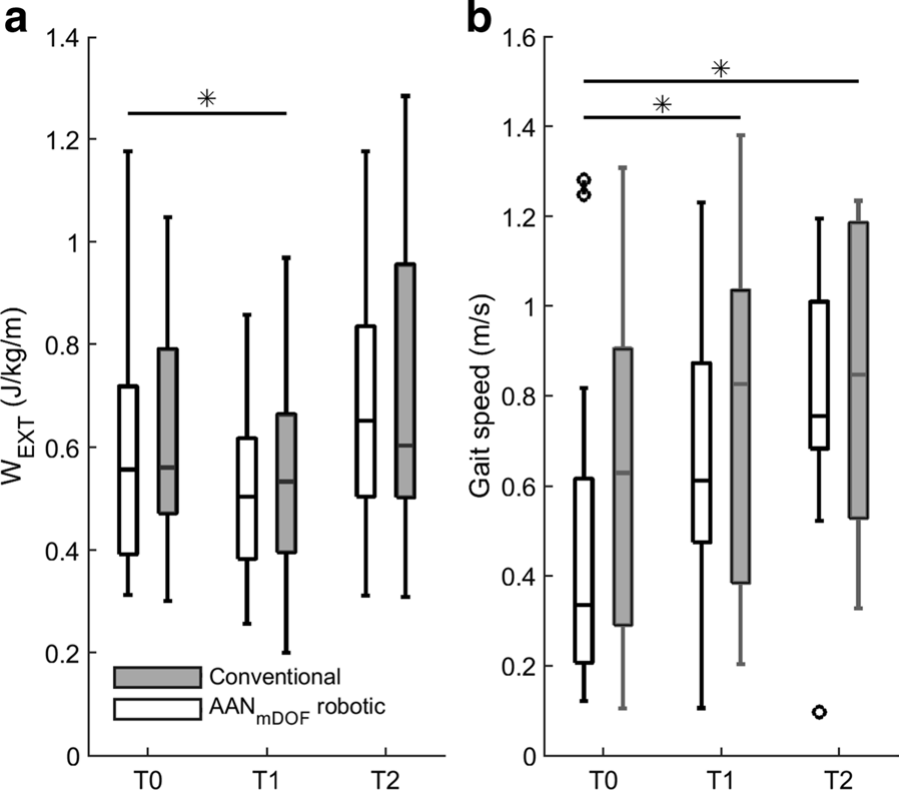
Course of change in **a** external mechanical work and **b** gait speed across assessments (T0–T2) in the AAN_mDOF_ robotic and conventional gait training groups. Each box represents the median, and upper and lower quartiles of the variable, with whiskers extended to the extreme values. Outliers are represented by markers. * significant *Time* effect (p < 0.05)

**Fig. 4:**
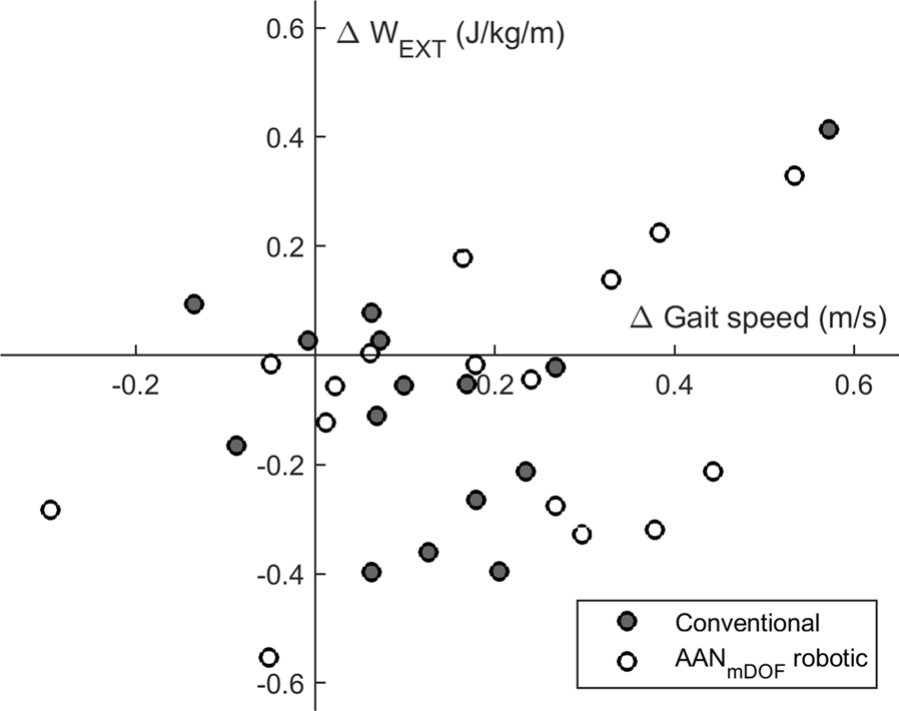
Individual change in gait speed plotted against the individual change in external mechanical work from T0 to T1, for individuals in the AAN_mDOF_ robotic and conventional gait training groups. Only data of individuals who completed both assessments at T0 and T1 are shown (n = 31). Positive change indicates an increased value of the variable at T1 relative to T0. Preferably, participants would be in the right lower quadrant (increased gait speed / decreased external work) or lower part of the right upper quadrant (increased gait speed / slightly increased external work)

Group results of W_EXT_ and gait speed are summarized in Table 2. The corresponding test statistics are reported in Additional file 1. Irrespective of group allocation (*Group* × *Time* interactions, p ≥ 0.438), W_EXT_ significantly decreased from T0 to T1 (mean difference = -0.09 J/kg/m; 95% CI − 0.17 to − 0.01, p = 0.039), while gait speed significantly increased from T0 to T1 (mean difference = 0.15 m/s; 95% CI 0.08–0.22, p < 0.001) (see Fig. 3). Figure 4 shows that 21 out of 31 participants who completed both assessments had lower W_EXT_ at T1. Seventeen of them (81%) showed a concurrent increase in gait speed, whereas four participants (19%) showed a concurrent decrease in gait speed. Of the 10 participants with increased W_EXT_ at T1, eight (80%) showed a concurrent increase and two (20%) a decrease in gait speed.

**Table 2:** Means (± SDs) of mechanical work, spatiotemporal gait parameters, functional gait tasks, and clinical scores for the AAN_mDOF_ robotic and conventional gait training groups, before (T0), immediately after (T1), and four months after (T2) the six-week intervention period

	AAN_mDOF_ robotic			Conventional		
	T0 n = 17	T1 n = 16	T2 n = 13	T0 n = 15	T1 n = 15	T2 n = 14
Mechanical work						
W_EXT_ (J/kg/m) *	0.61 ± 0.25	0.52 ± 0.18	0.69 ± 0.26	0.63 ± 0.23	0.53 ± 0.20	0.70 ± 0.29
Spatiotemporal parameters						
Gait speed (m/s) * **	0.47 ± 0.36	0.67 ± 0.29	0.81 ± 0.24	0.62 ± 0.36	0.75 ± 0.38	0.81 ± 0.32
Step width (m) * ^†^	0.15 ± 0.04	0.15 ± 0.05	0.14 ± 0.05	0.16 ± 0.06	0.18 ± 0.06	0.17 ± 0.06
Step length						
Paretic (m) * ** ^‡^	0.35 ± 0.15	0.46 ± 0.11	0.51 ± 0.10	0.43 ± 0.13	0.48 ± 0.13	0.49 ± 0.12
Non-paretic (m) * **	0.33 ± 0.17	0.44 ± 0.14	0.50 ± 0.09	0.39 ± 0.20	0.46 ± 0.19	0.50 ± 0.15
Symmetry ratio * **	0.04 ± 0.05	0.04 ± 0.03	0.03 ± 0.01	0.07 ± 0.10	0.04 ± 0.06	0.03 ± 0.03
Single-support time						
Paretic (% gait cycle) * **	0.28 ± 0.08	0.33 ± 0.06	0.34 ± 0.05	0.31 ± 0.07	0.33 ± 0.05	0.33 ± 0.04
Non-paretic (% gait cycle) *	0.32 ± 0.06	0.36 ± 0.05	0.36 ± 0.04	0.36 ± 0.05	0.37 ± 0.05	0.36 ± 0.04
Symmetry ratio * **	0.05 ± 0.05	0.03 ± 0.04	0.03 ± 0.03	0.04 ± 0.03	0.04 ± 0.03	0.03 ± 0.03
Functional gait tasks						
10-Meter Walk Test (m/s) * **	0.61 ± 0.35	0.86 ± 0.38	1.07 ± 0.27	0.76 ± 0.38	0.95 ± 0.38	1.07 ± 0.40
6-Minute Walk Test (m) * **	220 ± 149	301 ± 163	398 ± 119	247 ± 130	343 ± 147	383 ± 138
Functional Gait Assessment * **	14.7 ± 5.7	19.5 ± 4.8	23.6 ± 5.4	15.9 ± 5.8	21.7 ± 5.2	21.7 ± 5.3
Timed Up and Go test (s) * **	23.1 ± 15.0	17.0 ± 14.4	11.4 ± 6.1	19.5 ± 11.8	14.1 ± 7.6	12.4 ± 6.4
Clinical scores						
Fugl Meyer Assessment – leg score * **	24.2 ± 4.6	26.4 ± 5.0	28.9 ± 4.1	23.4 ± 6.8	28.9 ± 4.1	26.8 ± 5.5
Motricity Index – leg score * **	63.9 ± 17.0	77.0 ± 13.7	86.2 ± 13.2	62.5 ± 26.4	71.8 ± 24.6	72.5 ± 22.2

Functional Gait Assessment: range 0–30; Fugl Meyer Assessment – leg score: range 0–34; Motricity Index – leg score: range 0–100.

*significant *Time* effect T0 vs T1 (p ≤ 0.05); **significant *Time* effect T0 vs T2 (p ≤ 0.05); † significant *Group* x *Time* interaction T0 vs T1 (p ≤ 0.05); ‡ significant *Group* x *Time* interaction T0 vs T2 (p-value ≤ 0.05);

Between T0 and T2, W_EXT_ did not significantly differ (p = 0.263), while gait speed significantly increased in the same time period (mean difference = 0.26 m/s; 95% CI 0.18–0.34; p < 0.001) (see Fig. 3). These *Time* effects did not differ between groups (*Group × Time* interactions, p ≥ 0.152).

### Secondary outcomes

Paretic and non-paretic step length, paretic single-support time, step length and single-support time symmetry, and all functional gait tasks and clinical scores significantly improved from T0 to T1 (improvements ranging from 7.4 to 37.9%; p < 0.049) and T0 to T2 (improvements ranging from 14.5% to 67.6%; p < 0.019) (see Table 2 and Additional file 1). In addition, non-paretic single-support time improved from T0 to T1 (p = 0.005), and did not differ between T0 and T2 (p = 0.075). Most *Time* effects were similar for both groups from T0 to T1 (*Group* × *Time* interactions p ≥ 0.106), as well as from T0 to T2 (*Group* × *Time* interactions (p ≥ 0.063). From T0 to T1, the only significant difference between group was found for step width, which remained constant following robotic gait training, whereas it increased by 2 cm after conventional training (*Group* × *Time* interaction p = 0.018). Step width was similar for both groups between T0 to T2 (*Group* × *Time* interaction p = 0.055). Furthermore, from T0 to T2, the increase in paretic step length was larger following robotic gait training (16 cm) compared to conventional gait training (6 cm; *Group* × *Time* interaction p = 0.027). There were no main effects of *Group* for any outcome from T0 to T1 (p ≥ 0.152) or from T0 to T2 (p ≥ 0.201).

### Individual training goals

Participants with a pre-defined training goal aimed at foot clearance (n = 12) did not show a significant difference in the change in peak knee flexion between the robotic and conventional training group from T0 to T1 (p = 0.055), but this parameter reached significance in favor of the robotic training group when comparing T0 with T2 (p = 0.016, effect size *r* = 0.55) (see Table 3 and Additional file 2). Participants with a pre-defined training goal aimed at knee stability (n = 13) did not show significant differences in the change in maximal knee extension velocity of the paretic relative to the non-paretic leg between groups for either time interval (T0 vs T1, p = 0.570; T0 vs T2, p = 0.796). Six participants had a primary training goal aimed at improving limb loading and one participant at improving foot prepositioning. These subgroups were considered too small to allow statistical sub-analysis.

**Table 3:** Medians (ranges) of gait kinematics related to individual pre-defined training goals for the AAN_mDOF_ robotic and conventional gait training groups before (T0), immediately after (T1), and four months after (T2) the intervention period

	AAN_mDOF_ robotic			Conventional		
	T0	T1	T2	T0	T1	T2
Foot clearance	n = 6	n = 6	n = 6	n = 6	n = 6	n = 6
Peak knee flexion (°)**	31.6 (11.4–54.3)	47.9 (16.3–60.0)	52.4 (16.9–59.5)	42.1 (29.5–56.6)	36.5 (22.4–64.1)	33.9 (20.7–53.5)
Knee stability	n = 6	n = 5	n = 3	n = 7	n = 7	n = 6
Difference in paretic vs non-paretic maximum knee extension velocity (°/s)	− 13.7 (− 76.2–27.5)	− 9.0 (− 22.9–37.4)	− 20.9 (− 67.3–32.7)	− 25.6 (− 100.6–41.3)	− 34.6 (− 79.3–67.1)	− 7.5 (− 55.3–53.0)
Limb loading	n = 4	n = 4	n = 3	n = 2	n = 2	n = 2
Single-support time symmetry ratio	0.02 (0.01–0.15)	0.01 (0–0.14)	0.01 (0–0.13)	0.04 (0.04–0.04)	0.02 (0–0.03)	0.02 (0–0.04)

**Significant between group difference T0 vs T2 (p ≤ 0.05). The individual pre-defined training goal foot prepositioning was excluded from analysis because n = 1

## Discussion

Our hypothesis that, in the subacute phase after stroke, six weeks of AAN_mDOF_ robotic gait training would be superior to conventional gait training in terms of W_EXT_ (as a generic measure of the quality of the gait pattern) was not corroborated by the results of this study. Both the AAN_mDOF_ robotic and conventional gait training groups showed equally reduced W_EXT_ one week after the intervention period, combined with similarly increased gait speed. At four months follow-up, there was a further and similar increase in gait speed in both groups, while W_EXT_ returned to baseline values. In addition, compared to baseline, most spatiotemporal parameters, all functional gait tasks and all clinical scores had similarly improved in both groups one week after the intervention and at follow-up. The AAN_mDOF_ robotic gait training group showed no difference in step width one week after the intervention, in contrast to a slight increase in the conventional training group. In addition, at follow-up, paretic step length had increased only in the AAN_mDOF_ robotic gait training group. Furthermore, of all patients with a predefined goal aimed at foot clearance, only those who received AAN_mDOF_ robotic gait training were able to improve their maximal knee flexion after the intervention. No such subgroup differences were observed for patients with other predefined goals such as knee stability or limb loading.

Overall, our findings do not indicate a clear superior effect of AAN_mDOF_ robotic gait training compared to conventional gait training during primary inpatient stroke rehabilitation. Although the conventional gait training group showed a potentially undesirable increase in step width directly after the intervention period, the change was very small (2 cm) and step width at follow-up remained similar in both groups. Additionally, the AAN_mDOF_ robotic gait training group had increased their paretic step length at follow-up more than the conventional training group, but this effect was related to a shorter paretic step length at baseline in the robotic group. Indeed, both groups reached almost perfect symmetry at follow-up. Consequently, the clinical relevance of these findings is questionable. Hence, the data suggest that people after stroke recover in terms of motor impairments (clinical scores) and motor capacities (W_EXT_, gait speed, symmetry, and functional gait tasks) independent of the type of gait training. Our findings are in line with previous studies reporting beneficial effects of AAN_mDOF_ robotic gait training (*not* complemented with conventional gait training) on the over ground gait pattern and on clinical outcomes in chronic stroke survivors [44–46]. Furthermore, such AAN_mDOF_ robotic gait training combined with functional electrical stimulation was not found to be superior to therapist-assisted body-weight supported treadmill training in a small group of stroke survivors [45]. Hence, the findings of our randomized controlled trial add up to the current evidence that the effectiveness of AAN_mDOF_ robotic gait training is limited, but that AAN_mDOF_ robotic gait training might be used as an alternative for conventional gait training.

One week after the intervention, an increase in gait speed and concurrent decrease in W_EXT_ was observed in both groups. In contrast, previous studies have shown that faster gait speed is typically associated with increased levels of W_EXT_ [26, 30, 31]. In line, eight of our participants had increased their gait speed and increased their W_EXT_ accordingly (see Fig. 4). However, most of our participants (n = 17) showed an increased gait speed and a concurrent *decrease* in W_EXT_. This observed decrease in W_EXT_ while walking at a faster speed can be explained by reduced COM movements relative to the surroundings, suggesting that participants reduced their (compensatory) movements in the planes perpendicular to the walking direction. Taken together, these results indicate a more mechanically efficient, and better qualitative gait pattern one week after the intervention in both groups, which is supported by concurrent improvements in gait symmetry in both groups. Interestingly, at follow-up, the gait speed had further increased in both groups, however, now combined with a concurrent *increase* in W_EXT_ to baseline values. This suggests a further increase in functional gait capacity with a stabilization of mechanical efficiency and quality of the gait pattern in both groups four months after the intervention.

Although the analysis of individual training goals demonstrated mixed results, of all participants with a pre-defined goal aimed at improving foot clearance, only those who received robotic gait training had increased their peak knee flexion during swing at follow-up (+ 66%), whereas peak knee flexion had decreased at follow-up in those who received conventional gait training (− 19%) (see Table 3). Individuals in the conventional gait training group may have relied more on compensatory pelvic hike and hip abduction (‘circumduction’) to ensure foot clearance [5]. Although the effect size of this subgroup difference seems to be fairly large, the statistics are based on a small group size and, thus, should be interpreted with caution. It might be that individuals in the AAN_mDOF_ robotic gait training group benefited from appropriate proprioceptive information through continuous adaption of knee joint guidance from the AAN_mDOF_ robot. Therefore, AAN_mDOF_ robotic gait training that can support specific subtasks of the gait cycle seems to have the possibility to promote gait kinematics, but further research with larger group sizes is needed to determine its effect on all prerequisites of gait.

A limitation of the present study is that the generalizability of our results is limited to people suffering from primary or recurrent unilateral supratentorial stroke with independent ambulation prior to their stroke, a minimal level of independent ambulation after their stroke, and without relevant comorbidities. As a consequence, merely 7.5% of the individuals assessed for eligibility were eventually randomized to one of the training groups. Because participants had to be able to perform the gait analysis independently, individuals with poor (dependent) ambulatory capacity were excluded. As this latter group may typically profit from mechanically assisted gait training [47], it is still relevant to investigate the effect of AAN_mDOF_ robotic gait training in those with more severely affected gait capacity after stroke. A second limitation is that the study may lack sufficient power, as the number of included participants was smaller than the calculated sample size. Nevertheless, it should be noted that the original power calculation was based on a Beta of 10%. Using a Beta of 20% would have required 36 participants. Given the current sample size of 34 participants and the absence of any trend in the *Group* × *Time* interaction effects, we assume that the chance of false-negative study results is very small. A third limitation is that, with regard to the gait training, the AAN_mDOF_ robotic training group ultimately received 19% more training sessions than the conventional training group. This difference in training intensity might have worked in favor of the robotic group, but the results did not show any indication of such an effect. Lastly, the calculation of W_EXT_ was based on the COM movements derived from the gait kinematics instead of integrating ground reaction forces [48]. As it was difficult for several participants to successfully hit the force plate during gait analysis, ground reaction forces could not be recorded in a sufficient number of steps to be analyzed properly. Although the use of COM movements derived from kinematics implies multiple assumptions about anthropometry, rigidity of body segments, and correct marker placement, this method still appears to be valid for calculating W_EXT_ [49].

## Conclusion

AAN_mDOF_ robotic gait training was not superior to conventional gait training for improving W_EXT_, spatiotemporal gait characteristics, functional gait tasks, or clinical scores in stroke survivors during their primary inpatient rehabilitation. However, we found some indication of a beneficial (kinematic) effect of AAN_mDOF_ robotic gait training on peak knee flexion during the swing phase in a subgroup of participants with a predefined training goal aimed at improving foot clearance.

## Supplementary Information

**Additional file 1**. Test statistics—External work, gait speed and secondary outcomes.

**Additional file 2**. Test statistics—Individual training goals.

## Data Availability

The datasets used during the current study are available from the corresponding author on reasonable request.

## Abbreviations

AAN: Assist-as-needed
AAN_mDOF_: Assist-as-needed and multiple degrees of freedom
COM: Center of mass of the body
E_EXT_: Energy level of the body
W_EXT_: External mechanical work
FAC: Functional Ambulation Category
HADS: Hospital Anxiety and Depression Scale
MAS: Modified Ashworth Scale
MoCA: Montreal Cognitive Assessment
UCO: Utrechts Communicatie Onderzoek
IQR: Interquartile range
