# Supplementary material for: Effect of assist-as-needed robotic gait training on the gait pattern post stroke: a randomized controlled trial
Source: J Neuroeng Rehabil. 2021 Feb 5;18:26. doi: 10.1186/s12984-020-00800-4 (PMC7863532; doi:10.1186/s12984-020-00800-4)
Supplement: Supplementary file 1 — Additional file 1. Test statistics—External work, gait speed and secondary outcomes. [file 12984_2020_800_MOESM1_ESM.pdf]

Additional Table 1. Results of the mixed model analyses on both groups for two time intervals

	T0 vs T1			T0 vs T2		
	Time	Group	Interaction	Time	Group	Interaction
Mechanical work						
W_{EXT} (J/kg/m)	F(1,29.583) =4.680; p=0.039	F(1,29.864) =0.037; p=0.849	F(1,29.583) =0.008; p=0.930	F(1,27.835) =1.304; p=0.263	F(1,27.945) =0.018; p=0.895	F(1,27.835) =0.010; p=0.926
Spatiotemporal parameters						
Gait speed (m/s)	F(1,29.287) =20.015; p<0.001	F(1,30.015) =1.016; p=0.321	F(1,29.287) =0.619; p=0.438	F(1,28.365) =45.365; p<0.001	F(1,30.213) =0.681; p=0.416	F(1,28.365) =2.168; p=0.152
Step width (m)	F(1,29.113) =1.832; p=0.186	F(1,30.108) =0.602; p=0.444	F(1,29.113) =6.326; p=0.018	F(1,25.504) =0.013; p=0.910	F(1,30.256) =0.850; p=0.364	F(1,25.504) =4.058; p=0.055
Step length						
Paretic (m)	F(1,28.284) =29.077; p<0.001	F(1,29.570) =1.647; p=0.209	F(1,28.284) =2.504; p=0.125	F(1,26.721) =39.272; p<0.001	F(1,29.594) =0.958; p=0.336	F(1,26.721) =5.476; p=0.027
Non-paretic (m)	F(1,29.287) =41.377; p<0.001	F(1,29.972) =0.554; p=0.463	F(1,29.287) =0.594; p=0.447	F(1,28.695) =38.621; p<0.001	F(1,29.938) =0.521; p=0.476	F(1,28.695) =0.676; p=0.418
Symmetry ratio	F(1,29.564) =4.228; p=0.049	F(1,29.893) =0.565; p=0.458	F(1,29.564) =1.672; p=0.206	F(1,30.076) =6.1272; p=0.019	F(1,30.085) =0.876; p=0.357	F(1,30.076) =1.035; p=0.317
Single-support time						
Paretic (% gait cycle)	F(1,29.904) =20.695; p<0.001	F(1,30.098) =0.960; p=0.335	F(1,29.904) =2.786; p=0.106	F(1,25.735) =27.566; p<0.001	F(1,29.148) =0.809; p=0.376	F(1,25.735) =3.768; p=0.063
Non-paretic (% gait cycle)	F(1,29.590) =9.5382; p=0.005	F(1,30.114) =2.164; p=0.152	F(1,29.590) =1.633; p=0.211	F(1,29.496) =3.408; p=0.075	F(1,30.098) =1.711; p=0.201	F(1,29.496) =2.210; p=0. 148
Symmetry ratio	F(1,29.925) =6.471; p=0.016	F(1,30.114) =0.032; p=0.859	F(1,29.925) =3.819; p=0.060	F(1,25.113) =7.102; p=0.013	F(1,28.395) =0.120; p=0.732	F(1,25.113) =0.217; p=0.645
Functional gait tasks						
10-Meter Walk Test (m/s)	F(1,29.043) =49.900; p<0.001	F(1,29.950) =1.066; p=0.310	F(1,29.0.43) =0.811; p=0.375	F(1,25.658) =81.039; p<0.001	F(1,29.998) =1.101; p=0.302	F(1,25.658) =0.675; p=0.419
6-Minute Walk Test (m)	F(1,29.038) =59.246; p<0.001	F(1,29.959) =0.585; p=0.450	F(1,29.038) =1.367; p=0.252	F(1,25.332) =111.055; p<0.001	F(1,29.782) =0.187; p=0.668	F(1,25.332) =0.197; p=0.661
Functional Gait Assessment	F(1,29.053) =61.375; p<0.001	F(1,29.834) =1.016; p=0.321	F(1,29.053) =1.029; p=0.319	F(1,26.189) =78.709; p<0.001	F(1,30.034) =0.000; p=0.991	F(1,26.189) =2.156; p=0.154
Timed Up and Go test (s)	F(1,28.779) =23.613; p<0.001	F(1,29.683) =0.792; p=0.381	F(1,28.779) =0.112; p=0.740	F(1,23.328) =31.287; p<0.001	F(1,27.521) =0.663; p=0.422	F(1,23.328) =0.076; p=0.785
Clinical scores						
Fugl Meyer Assessment – leg score	F(1,29.225) =19.525; p<0.001	F(1,30.088) =0.109; p=0.743	F(1,29.225) =0.095; p=0.760	F(1,25.070) =27.119; p<0.001	F(1,28.953) =0.303; p=0.586	F(1,25.070) =0.080; p=0.780
Motricity Index – leg score	F(1,29.065) =23.784; p<0.001	F(1,29.892) =0.090; p=0.767	F(1,29.065) =0.121; p=0.731	F(1,25.579) =35.326; p<0.001	F(1,29.727) =0.610; p=0.441	F(1,25.579) =2.439; p=0.131
