# Supplementary material for: Effect of assist-as-needed robotic gait training on the gait pattern post stroke: a randomized controlled trial
Source: J Neuroeng Rehabil. 2021 Feb 5;18:26. doi: 10.1186/s12984-020-00800-4 (PMC7863532; doi:10.1186/s12984-020-00800-4)
Supplement: Supplementary file 2 — Additional file 2. Test statistics—Individual training goals. [file 12984_2020_800_MOESM2_ESM.pdf]

Additional Table 2. Results of the Mann-Whitney U analyses of gait kinematics related to individual pre-defined training goals in both groups for two time intervals

	T0 vs T1	T0 vs T2
Foot clearance		
Peak knee flexion during the swing phase	U=6.000; z=-1.922; p=0.055	U=3.000; z=-2.402; p=0.016
Knee stability		
Difference in paretic vs non-paretic maximum knee extension velocity during the stance phase	U=14.000; z=-0.568; p=0.570	U=8.000 ;z=-0.258; p=0.796
